# Patterns of care and clinical outcome in assumed glioblastoma without tissue diagnosis: A population-based study of 131 consecutive patients

**DOI:** 10.1371/journal.pone.0228480

**Published:** 2020-02-13

**Authors:** Katja Werlenius, Boglarka Fekete, Malin Blomstrand, Helena Carén, Asgeir S. Jakola, Bertil Rydenhag, Anja Smits

**Affiliations:** 1 Department of Oncology, Sahlgrenska University Hospital, Gothenburg, Sweden; 2 Department of Oncology, Institute of Clinical Sciences, Sahlgrenska Academy, University of Gothenburg, Gothenburg, Sweden; 3 Department of Clinical Neuroscience, Institute of Neuroscience and Physiology, Sahlgrenska Academy, Gothenburg, Sweden; 4 Sahlgrenska Cancer Center, Department of Laboratory Medicine, Institute of Biomedicine, Sahlgrenska Academy, University of Gothenburg, Gothenburg, Sweden; 5 Department of Neurosurgery, Sahlgrenska University Hospital, Gothenburg, Sweden; 6 Department of Neurology, Sahlgrenska University Hospital, Gothenburg, Sweden; 7 Department of Neuroscience, Neurology, Uppsala University, Gothenburg, Sweden; George Washington University, UNITED STATES

## Abstract

**Background:**

Elderly patients with glioblastoma and an accumulation of negative prognostic factors have an extremely short survival. There is no consensus on the clinical management of these patients and many may escape histologically verified diagnosis. The primary aim of this study was to characterize this particular subgroup of patients with radiological glioblastoma diagnosis without histological verification. The secondary aim was to evaluate if oncological therapy was of benefit.

**Methods:**

Between November 2012 and June 2016, all consecutive patients presenting with a suspected glioblastoma in the western region of Sweden were registered in a population-based study. Of the 378 patients, 131 (35%) met the inclusion criteria of the present study by typical radiological features of glioblastoma without histological verification.

**Results:**

The clinical characteristics of the 131 patients (72 men, 59 women) were: age ≥ 75 (n = 99, 76%), performance status according to Eastern Cooperative Oncology Group ≥ 2 (n = 93, 71%), significant comorbidity (n = 65, 50%) and multilobular tumors (n = 90, 69%). The overall median survival rate was 3.6 months. A subgroup of 44 patients (34%) received upfront treatment with temozolomide, with an overall radiological response rate of 34% and a median survival of 6.8 months, compared to 2.7 months for those receiving best supportive care only. Good performance status and temozolomide treatment were statistically significant favorable prognostic factors, while younger age was not.

**Conclusion:**

Thirty-five percent of patients with a radiological diagnosis of glioblastoma in our region lacked histological diagnosis. Apart from high age and poor performance status, they had more severe comorbidities and extensive tumor spread. Even for this poor prognostic group upfront treatment with temozolomide was shown of benefit in a subgroup of patients. Our data illustrate the need of non-invasive diagnostic methods to guide optimal individualized therapy for patients considered too fragile for neurosurgical biopsy.

## Introduction

Glioblastoma (GBM) is the most common malignant brain tumor in adults, constituting 46% of malignant primary brain tumors [1]. The incidence of GBM is known to increase with age [2], and GBM rates have successively increased over the last decades in the ageing population [2–6]. Elderly patients with GBM more often present with poor performance status (PS) and significant comorbidities. At the same time, high age is amongst the strongest unfavorable prognostic factors for survival [7–11], and benefits of treatment are small compared to the younger population with GBM [10]. Furthermore, treatment seems to be tolerated worse amongst the elderly [12, 13]. A higher risk of postoperative complications in frail and elderly patients has been reported [14, 15], as well as shorter survival for those suffering from complications after surgery [14, 16].

As a consequence, elderly patients with an unambiguous radiological diagnosis of GBM and an accumulation of negative prognostic factors at disease presentation may receive best supportive care (BSC) without histological verification of tumor diagnosis [9, 17–19]. The exact proportion of this subgroup in the general GBM population is not well known, but numbers varying between 8–19% have been reported from national brain tumor registries [9, 17, 20, 21]. When taking the elderly population into consideration exclusively, the proportion of patients receiving BSC only has been reported as high as 44–75% [10, 17, 19, 22].

It is not clear whether there is a general underreporting of patients lacking histological tumor diagnosis or whether the variations in reported numbers represent true differences in clinical strategies between different centers and countries. In conclusion, the subgroup of elderly and fragile patients with GBM has largely gone unstudied. At the same time, these patients represent an important part of everyday clinical practice that can neither be ignored nor be expected to diminish over time.

Until recently, the Swedish Brain Tumor Registry included patients with histologically verified brain tumor diagnoses only. Thus, this registry cannot be used to study the particular subgroup of patients without tissue diagnosis. In our previous population-based study from the western region of Sweden, we found that 47% of all patients with a typical radiological diagnosis of GBM during 2004–2008 were not considered for diagnostic surgery [19]. As expected, the survival for this unfavorable prognostic subgroup of untreated GBM was extremely poor, with a median overall survival of only 3.2 months, illustrating the aggressive natural course of the disease.

In the present study, we characterized the subpopulation of patients with radiological diagnosis of GBM without histological verification during 2012–2016, with focus on clinical characteristics, patterns of care and outcome. We also evaluated if there has been a change in practice over time and if oncological treatment benefitted this poor prognostic group of patients.

## Patients and methods

### Patients

Between November 2012 and June 2016, all adult patients (≥ 18 years) presenting with a suspected radiological diagnosis of supratentorial GBM in the western health care region of Sweden (approx. 1.9 million inhabitants) were registered in a population-based consecutive study [23]. There is one single neurosurgical department serving the entire health care region, and no private health care for patients with brain tumors, assuring identification of all patients presenting with suspected GBM in the region during the study period.

All patients were discussed at the multidisciplinary neuro-oncological tumor board (MTB) at Sahlgrenska University Hospital, Gothenburg, and individual treatment recommendations were documented in the medical records and the meeting protocols.

**Fig 1:**
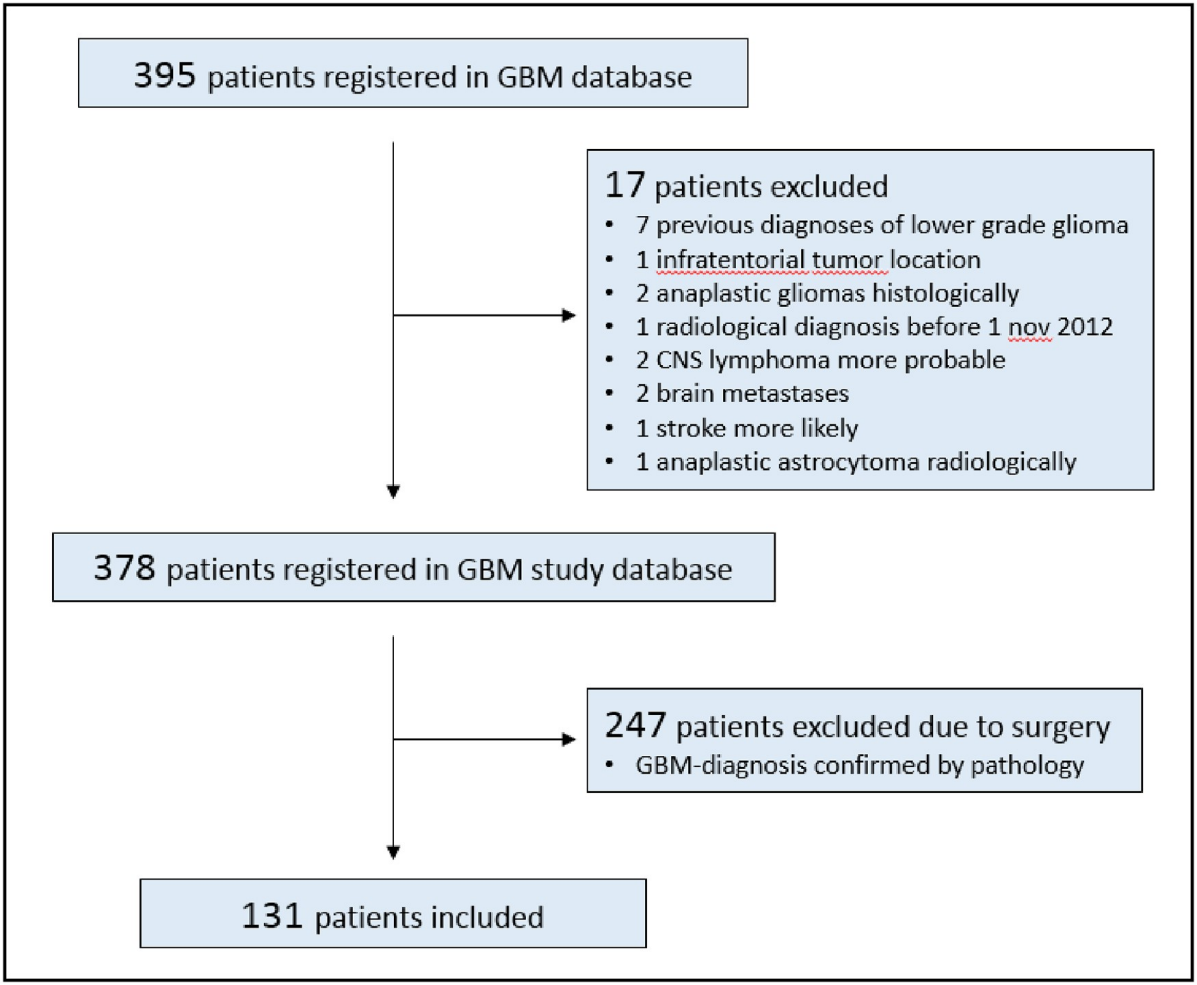
Flow chart illustrating the patient selection in the present study.

Of the in total 395 patients with radiological diagnosis of GBM registered in the data base, 148 patients were not recommended for surgery. The specific motivations to refrain from surgery (such as age, tumor location and spread, comorbidity, PS, previous histological diagnosis of lower-grade glioma or the individual wish of the patient) were documented. A retrospective review of the medical charts of these patients together with a re-evaluation of diagnostic imaging was performed for the purpose of this study. Following this evaluation, 131 patients (35%) met the inclusion criteria of the present study by typical radiological features of GBM without histological verification (Fig 1).

### Clinical characteristics

All patients were followed for survival until death or the end of the study (30 June 2018). The following data were collected from medical records: date of diagnosis (i.e. date of the first brain scan showing typical features of GBM), type of diagnostic imaging, age at diagnosis, sex, tumor location, presenting symptoms, comorbidity, estimated PS according to Eastern Cooperative Oncology Group (ECOG) [24] at diagnosis, and treatment (supportive care or oncological treatment)). Multilobular tumors were defined as involving two or more cerebral lobes, multifocal as at least two separate contrast-enhancing lesions. Comorbidities were scored according to an adapted Charlson Comorbidity Index (CCI) [25], using the assigned weights for diseases modified by Schneeweiss [26]. Significant comorbidity was defined as a score of ≥ 2.

For patients receiving oncological treatment, the following additional data were registered: start- and stop-dates for oncological treatment, PS at follow-up, use of corticosteroids at start of treatment, type and number of oncological therapies, radiological response to treatment, clinical benefit and bone marrow toxicity (defined as platelets <100 x10^9^/L or total white blood count <3.0 x10^9^/L).

### Statistical analysis

Descriptive statistics were used for patient characteristics and oncological treatment. Statistical calculations were performed using IBM SPSS Statistics version 25 (IBM cooperation, Armonk, NY, USA). Differences in distribution of clinical variables between the BSC group and the group of patients receiving oncological treatment were calculated by independent samples t-test for continuous variables and Fisher exact test for proportions. All p-values were two-sided. Survival was estimated by the Kaplan-Meier method and Log-rank test. A p-value of <0.05 was considered statistically significant.

### Ethical review process

Approval for inclusion of patients in the database was obtained by the Ethics committee (Regional Ethics Review Board, Dnr 559–12, date of approval 2012-08-15). For inclusion of patients (without histological diagnosis) in the present study, an amendment was submitted and approved (Regional Ethics Review Board, Dnr 505–18, date of approval 2018-05-30). For this particular part of the study, the ethics committee waived the requirements for informed consent based on the fact that all patients to be included were deceased at that time point. Patients’ medical records were obtained from the Sahlgrenska University Hospital, Gothenburg and analyzed during the time period May 2018—April 2019. All data were fully anonymized before they were accessed for the purpose of the study.

## Results

### Patient characteristics

The clinical characteristics of the 131 patients are listed in Table 1. As shown, the median age at diagnosis was 79.7 years (range 52.8–90.5). A total of 99 patients (76%) were ≥ 75 years, 72 men (55%) and 59 women (45%), with a male-to-female ratio of 1.22. At the time of diagnosis, 93 patients (71%) had PS ≥ 2. Sixty-five patients (50%) had significant comorbidity (CCI ≥ 2) and 90 patients (69%) had multilobular tumors. In 90% of cases, radiological diagnosis was based on MRI with contrast-enhancement, three patients (2%) had MRI or CT without contrast-enhancement, due to individual contraindications.

**Table 1:** Baseline characteristics of patients with radiological diagnosis of GBM without histological confirmation.

Variable:	Number of patients (N = 131)
	N (%)
Age at diagnosis:	
< 60 years	5 (4)
60–74 years	27 (21)
≥ 75 years	99 (75)
Median age in years (range):	79.7 (52.8–90.5)
Gender:	
Male	72 (55)
Female	59 (45)
Performance status^1^:	
0	7 (5)
1	31 (24)
2	44 (34)
3	32 (24)
4	17 (13)
Tumor location:	
Right hemisphere	46 (35)
Left hemisphere	41 (31)
Bilateral	44 (34)
Multilobular tumor^2^:	90 (69)
Multifocal tumor^3^:	49 (37)
Method of diagnosis:	
MRI with contrast	118 (90)
CT with contrast	10 (8)
MRI or CT without contrast	3 (2)
Charlson Comorbidity Index^4^:	
0	37 (28)
1	29 (22)
2	22 (17)
3	21 (16)
≥4	22 (17)

^1^Performance status, according to ECOG, Eastern Cooperative Oncology Group/WHO,

^2^Multilobular tumor, involving two or more cerebral lobes,

^3^Multifocal tumor, defined as at least two separate contrast-enhancing lesions,

^4^ Comorbidity according to adjusted Charlson Comorbidity index

The most common motivations for not recommending diagnostic surgery by the MTB were extensive tumor infiltration, together with high age, eloquent areas, poor PS, and/or significant comorbidities. In three cases, diagnostic surgery was recommended by the MTB but not performed due to the individual wish of the patient. For comparative reasons, the main clinical characteristics of the cohort of patients undergoing diagnostic surgery for GBM (n = 247) during the study period are presented in the supporting information (S1 Table).

### Treatment characteristics

Fifty-four of the 131 patients (41%) were seen by an oncologist in the outpatient setting, and a total of 45 patients (34%) received oncological treatment. The clinical characteristics for patients receiving treatment as compared to BSC are shown in Table 2. As illustrated, patients who received treatment were generally younger, had better PS, less comorbidities, and more often multifocal tumors, while there was no difference in gender or tumor location. The vast majority (93%) of the 45 patients receiving treatment had corticosteroids at baseline. TMZ as a single agent was the most common first line treatment (n = 44, 98%). In total, three patients received a short-course of radiotherapy. Five out of the seven patients receiving second line treatment received lomustine as a single agent, as depicted in Table 3.

**Table 2:** Clinical characteristics for patients with radiological GBM receiving oncological treatment vs best supportive care.

Variable:	Number of patients (N = 131):		
	Oncological treatment N = 45 (34%)	Best Supportive Care N = 86 (66%)	p-value (χ^2^):
	N (%)	N (%)	
Age at diagnosis:			<0.0001*
< 75 years	24 (53)	8 (9)	
≥ 75 years	21 (47)	78 (91)	
Mean age (years ± SD):	72.7±8.0	81.5±5.2	<0.0001*
Gender:			0.3
Male	22 (49)	50 (58)	
Female	23 (51)	36 (42)	
Performance status^1^:			<0.0001*
0–1	23 (51)	15 (17)	
2–4	22 (49)	71 (83)	
Tumor location:			0.8
Unilateral	28 (62)	59 (69)	
Bilateral	17 (38)	27 (31)	
Multilobular^2^:	29 (64)	61 (71)	0.4
Multifocal^3^:	22 (49)	27 (31)	0.05*
Comorbidity^4^:			0.02*
0–1	29 (64)	37 (43)	
≥ 2	16 (36)	49 (57)	
Median survival in months (95% CI):	6.8 (5.6–8.0)	2.7 (2.4–3.1)	<0.0001*

^1^Performance status, according to ECOG,

^2^Multilobular tumor, involving two or more cerebral lobes,

^3^Multifocal tumor, defined as at least two separate contrast-enhancing lesions,

^4^According to adjusted Charlson Comorbidity Index,

*significant

**Table 3:** Treatment characteristics of patients without histopathological confirmation receiving oncological treatment.

	Number of patients (N = 45):
	N (%)
First line treatment:	
TMZ	44 (98)
Short course RT	1 (2)
Any second line treatment:	7 (16)
Treatment response TMZ:	(N = 44)
Radiological regression	15 (34)
Clinical benefit^1^	19 (43)
Bone marrow toxicity^2^:	10 (23)

^1^As decided by stable (PS ≤ 2) or improved PS after 3 months,

^2^White blood cell count <3.0x10^9^/L or platelets <100x10^9^/L

### Response to treatment and toxicity

**Fig 2:**
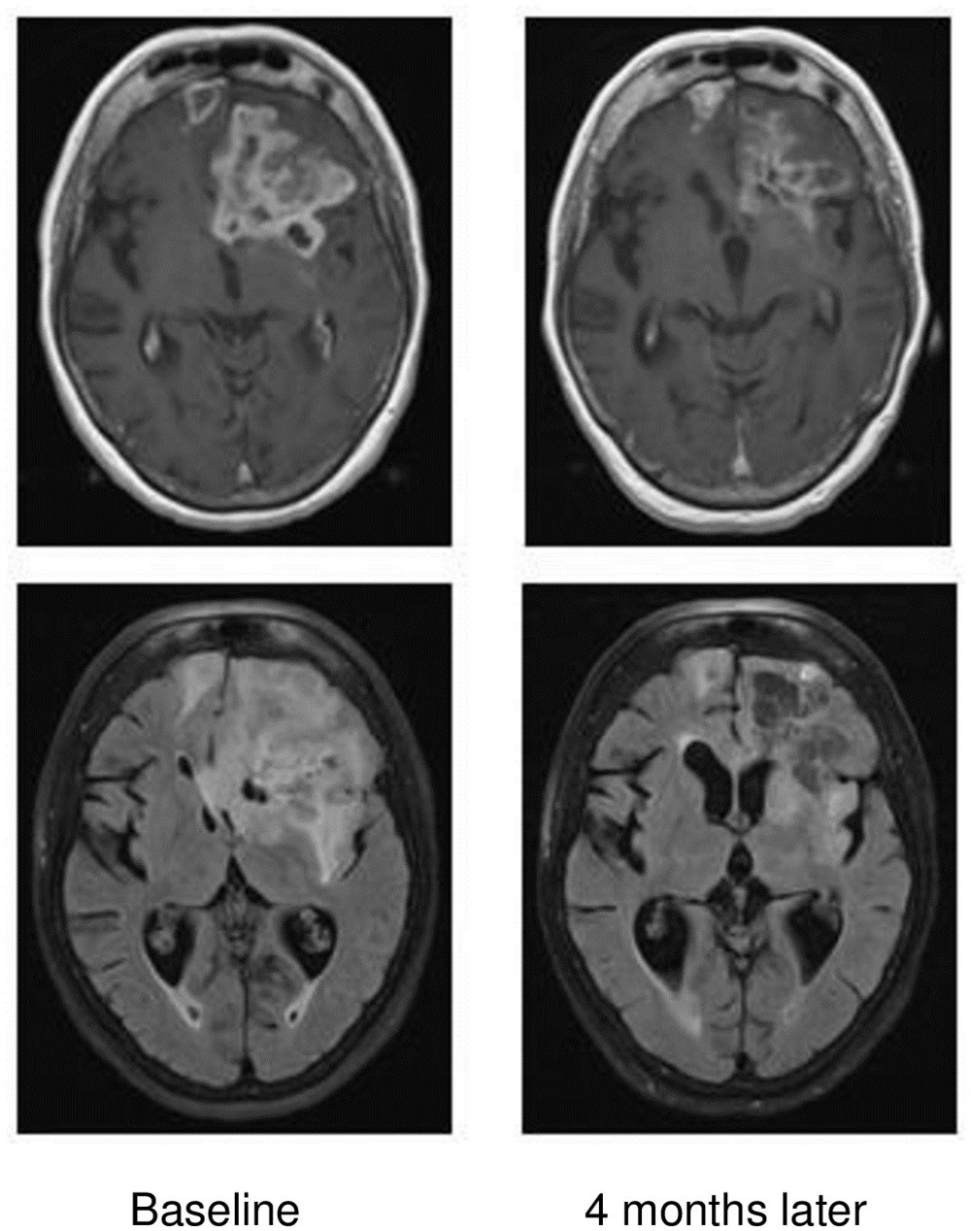
Illustrative case report. 73-year old patient who presented with confusion and change of personality. Diagnostic surgery was not considered suitable due to poor PS. The patient received palliative treatment with steroids and temozolomide, and responded radiologically and clinically, with improvement of cognition and performance status (PS 2 to PS 1), following three cycles of temozolomide treatment. The patient died 11.2 months after radiological diagnosis of glioblastoma.

The overall radiological response rate to TMZ was 34%. Nineteen patients (43%) remained stable or improved in PS at three months after chemotherapy (see case illustration, Fig 2). Ten patients experienced bone marrow toxicity, defined as white blood cell count <3.0 x10^9^/L or platelet count <100 x10^9^/L (Table 3). Three patients experienced thrombocytopenia grade 3 according to CTCAE (Common Terminology Criteria for Adverse Events), with platelet count in the range of 25–49 x10^9^/L. No grade 4 or 5 toxicities were observed.

### Survival

**Fig 3:**
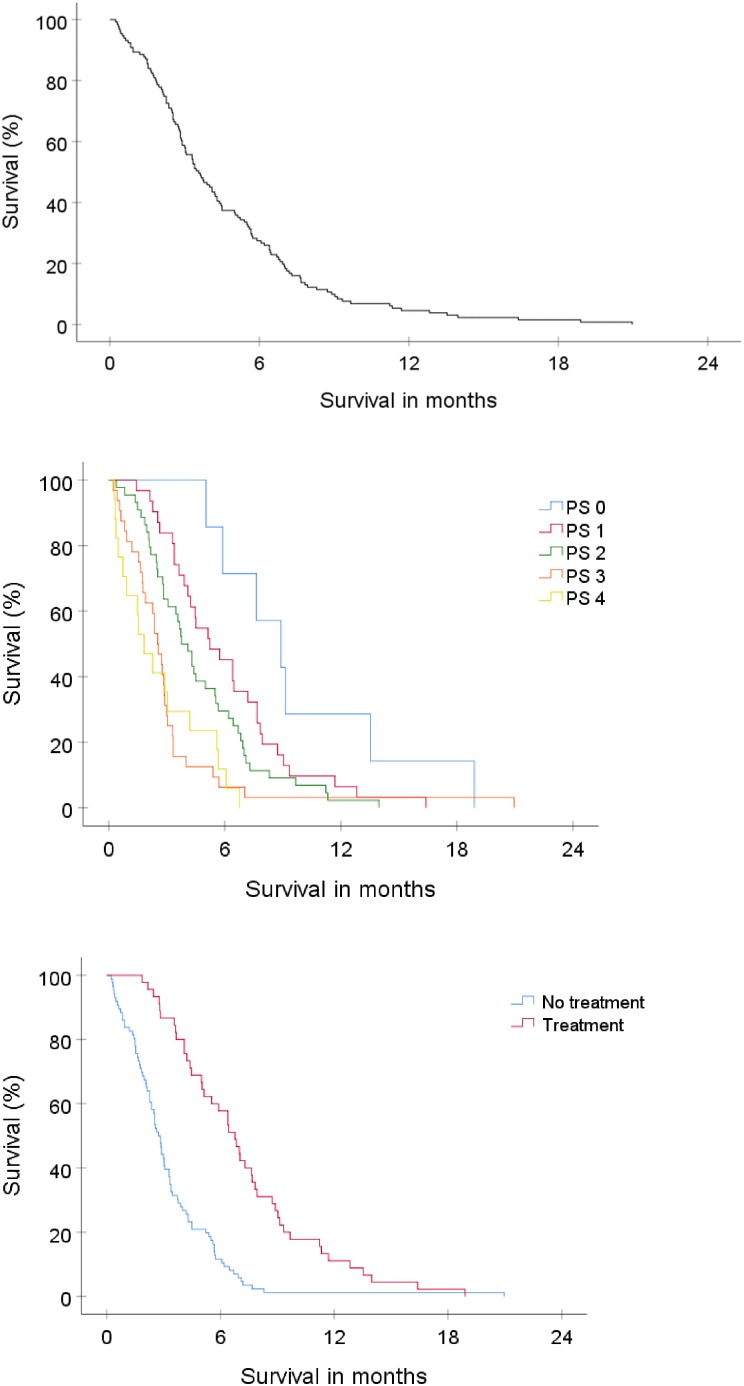
**a.** Overall survival for all patients with histologically unverified GBM. **b.** Survival stratified for performance status according to ECOG. **c.** Survival for patients receiving oncological treatment vs best supportive care only.

At the end of the study all patients had died. The median total survival in the whole group (n = 131) was 3.6 months (95% CI, 2.8–4.3). Survival rate at 6 and 12 months was 27% and 5% respectively. The median survival for patients receiving oncological treatment was 6.8 months (95% CI, 5.6–8.0) compared to 2.7 months (95% CI, 2.4–3.1) for patients receiving BSC (Fig 3a–3c).

### Predictors of survival

Univariate analysis by log-rank test identified PS and oncological treatment as the only significant predictors of survival (PS, p < 0.0001; oncological treatment, p < 0.0001; Fig 3b and 3c). Age (as dichotomized parameter with cut-off at 75, and as continuous parameter using Cox-regression), comorbidity, gender, tumor location and multifocality did not have any significant impact on survival.

Regression analysis with the aim to identify predictors of survival was not considered meaningful due to the strong association between the two variables PS and oncological treatment (Pearson Chi-Square p<0.0001), and this was considered a violation of the assumption of independence.

## Discussion

In the present study we describe a poor prognostic subgroup of patients with radiological but not histologically verified GBM diagnosis. The median age of these patients was significantly higher (79.7 years) compared to the general population of GBM (64 years), as recently reported by the CBTRUS [1]. In addition to high age, the majority (71%) of patients had a poor PS at the time of diagnosis. Poor PS is a well-known risk factor associated with a shorter life expectancy [7, 8, 11, 12]. Apart from high age and poor PS, individual motivations for refraining from diagnostic surgery were severe comorbidities and extensive tumor spread.

Interestingly, higher age was not associated with worse outcome in our study representing a selection of the very elderly GBM population. In contrast, we identified poor PS (ECOG ≥ 2) as a strong prognostic factor for shorter survival. Thus, for this selected subpopulation of GBM patients with poor outcome, PS was more important than factual age. We also found a less marked male predominance in our study compared to other reports [4]. This may be related to the fact that women in Sweden have a longer life-expectancy than men, but biological factors may also play a role in gender-related differences in the oldest age group.

Previous studies have shown that elderly patients are more likely to receive a GBM diagnosis without histological verification [3, 17, 20]. To our knowledge, the clinical characteristics and the outcome of this poor prognostic group have not been investigated earlier. As mentioned, the proportion of these patients in the general GBM population varies considerably between studies, suggesting differences in treatment strategies as well as in the registration of patients. In a previous population-based study from our own region during the time period 2004–2008, we found that 47% of patients lacked histological diagnosis [19], which is in line with a recent population-based British study of GBM patients aged >70 years [22]. In the present study, this proportion has decreased from 47% to 35%, while the median age of patients has increased to almost 80 years compared to 75 years in our previous cohort. These results indicate a shift in towards a more active policy for more fragile elderly patients with GBM in our region.

Although it is not standard of care to give oncological treatment without tissue diagnosis, it can be argued that refraining from surgery in patients with such a short life expectancy will decrease the likelihood for receiving no tumor treatment at all. Particularly elderly patients with poor preoperative PS seem to be at risk for postoperative complications [7, 8, 12]. This is probably the reason why up to 34% of patients do not receive oncological treatment after diagnostic biopsy [19, 22]. An even higher number was reported in an Australian study of GBM in patients aged 80 or older, where only 6 out of 40 patients continued with postoperative radiotherapy or chemotherapy [27].

Studies on elderly patients with GBM and poor PS are scarce, also because these patients are usually excluded from clinical trials. One important exception was provided by the French nonrandomized phase II trial evaluating the efficacy of TMZ in patients >70 years with newly diagnosed GBM and a poor postoperative PS [28]. More recently, a prospective phase III-study by Roa et al demonstrated that a one-week course of radiotherapy was non-inferior to a 3-week course of radiotherapy in elderly and/or frail patients [29].

In the present cohort, we used TMZ as a single agent instead of radiotherapy in almost all cases. Although not evidence-based, this strategy has been common practice in our region for elderly and frail patients with unknown *MGMT* methylation status and extensive tumor spread. Given the limited life expectancy of these patients, our experience is that the rapid start of TMZ treatment is an important advantage when waiting-time and hospitalization in connection with diagnostic surgery and radiotherapy can be detrimental, leaving no time left to benefit from treatment [14]. Indeed, we found that forgoing biopsy/resection reduced the waiting-time from radiological diagnosis to start of first oncological treatment by more than 50% (median of 28 days versus 59 days following diagnostic surgery) (S1 Table).

Several trials support an active treatment of elderly GBM patients with good PS [30–33], and it is well-established that high age per se, without poor PS or significant comorbidities, is not a contraindication for active treatment. Keime-Guibert et al demonstrated that radiotherapy (50 Gy in 28 fractions) was superior to best supportive care (BSC) with a median survival of 7 months for patients receiving radiotherapy compared to 4 months in the group receiving BSC only, but there was no observed improvement of health-related quality of life during or after radiotherapy [30]. The Nordic trial showed that TMZ as a single agent and short course RT (34 Gy in 3.4 fractions) was superior to long course radiotherapy (60 Gy in 30 fractions) for patients > 70 years [31]. For patients with hypermethylated *MGMT*, TMZ alone rendered the longest median survival of 9.7 months. Median age at diagnosis in the Nordic trial was 70 years, and more than 75% had good PS (ECOG 0–1). Another important observation from this trial was that patients receiving TMZ alone generally reported better health-related quality of life than patients receiving radiotherapy [31].

Recently, Perry et al demonstrated that the addition of TMZ to short course radiotherapy (40 Gy in 15 fractions) improved survival compared to radiotherapy alone [32]. This was particularly true for patients with hypermethylated *MGMT*, for whom median survival was 13.5 months compared to 7.7 months with radiotherapy alone. Interestingly, there was a survival benefit of 2.1 months, although not statistically significant (P = 0.055), for patients with unmethylated *MGMT* as well. The median age of the study population was 73 years, with almost 30% of the patients > 75 years old, and 77% with PS 0 or 1 [32].

Our study showed a significant better survival for patients receiving oncological treatment compared to patients receiving BSC (6.8 vs 2.7 months). Obviously, no firm conclusions can be drawn since there was a strong selection bias for patients receiving treatment based on PS and age. The better outcome of the treated group is, however, consistent with the results of the previously mentioned French phase II trial, evaluating TMZ after surgery (biopsy 91%) in patients >70 years with newly diagnosed GBM and a poor postoperative PS. Median OS in the French trial was 6.2 months and 33% of the patients improved their functional status, demonstrating that elderly frail patient may benefit from TMZ alone [28]. A meta-analysis found TMZ to be non-inferior to radiotherapy for elderly patients, particularly for those with hypermethylated *MGMT* [34]. In another systematic review it was concluded that elderly patients with *MGMT* promotor methylated tumors who are not candidates for combined radiochemotherapy, are more likely to benefit from TMZ alone than radiotherapy [35]. In a longer perspective, these data strongly suggest that the old and frail GBM population would benefit from new non-invasive diagnostic methods prior to individual treatment decisions. Blood-based liquid biopsy has emerged as a promising resource not only for initial diagnosis but also for prognostic prediction, treatment planning and follow-up [36].

An obvious limitation of our study, which is intrinsic to the study design, is the lack of a definite GBM diagnosis confirmed by operation or autopsy, implying that some of the patients in the cohort might actually not have a GBM. However, for all 131 patients the clinical course of disease was in total agreement with GBM diagnosis.

It cannot be excluded that refraining from diagnostic surgery has limited the treatment options and thereby influenced the outcome of individual cases. As such, it is possible that some patients with better PS would have benefitted from surgery and a more active oncological treatment approach with combined radiation and chemotherapy. On the other hand, others may never have come to treatment due to postoperative complications and poor PS following surgical intervention.

## Conclusions

We report that 35% of all patients with radiological diagnosis of GBM in the western region of Sweden lacked histological diagnosis. This group consisted mainly of elderly patients with poor PS, significant comorbidity and extensive tumor spread, and had a median survival of 3.6 months. The median survival in a subgroup of patients receiving single treatment with TMZ was 6.8 months compared to 2.7 months for those receiving best supportive care only. Our results suggest that even in this poor prognostic group upfront treatment with TMZ may be of benefit. Our data illustrate the need of novel non-invasive diagnostic methods for patients considered too fragile for neurosurgical biopsy to guide tailored therapy.

## Supporting information

S1 Table Clinical characteristics of GBM patients in the western region of Sweden between November 2012 and June 2016. (DOCX)

S2 Table De-identified minimal data set of the study population. (DOCX)
