# Supplementary material for: Patterns of care and clinical outcome in assumed glioblastoma without tissue diagnosis: A population-based study of 131 consecutive patients
Source: PLoS One. 2020 Feb 13;15(2):e0228480. doi: 10.1371/journal.pone.0228480 (PMC7017992; doi:10.1371/journal.pone.0228480)
Supplement: S1 Table — (DOCX) [file pone.0228480.s001.docx]

| **S1 Table** | | | | | | | |  |  |  |  |
| --- | --- | --- | --- | --- | --- | --- | --- | --- | --- | --- | --- |
| Clinical characteristics of all glioblastoma patients in the western region of Sweden between November 2012 and June 2016 | | | | | | | | | | | |
| Variable: |  | Number of patients diagnosed by: | | | | | | | | | |
| Radiology (N=131)  N (%) | | | Histopathology (N= 247)  N (%) | | | p-value | | | |  |  |
| Age at diagnosis: | |  |  |  |  |  |  |  | <0.0001* | |  |
| < 75 years | |  | 32 (24) |  |  | 229 (93) |  |  |  | |  |
| ≥ 75 years | |  | 99 (76) |  |  | 18 (7) |  |  |  | |  |
| Median age (years and range): | | | 79.7 (52.8-90.5) | |  | 62.5 (19.4-82.2) | |  | <0.0001* | |  |
| Performance status^1^: | | |  |  |  |  |  |  | <0.0001* | |  |
| 0-1 | |  | 38 (29) |  |  | 178 (72) |  |  |  | |  |
| ≥ 2 | |  | 93 (71) |  |  | 69 (28) |  |  |  | |  |
| Tumor location: | |  |  |  |  |  |  |  | <0.0001* | |  |
| Unilateral | |  | 87 (66) |  |  | 229 (93) |  |  |  | |  |
| Bilateral | |  | 44 (34) |  |  | 18 (7) |  |  |  | |  |
| Multilobular^2^ | |  | 90 (69) |  |  | 119 (48) |  |  | <0.0001* | |  |
| Multifocal^3^ | |  | 49 (37) |  |  | 57 (23) |  |  | 0.003* | |  |
| Oncological treatment: | | |  |  |  |  |  |  | <0.0001* | |  |
| No: | | | 86 (66) |  |  | 16 (6) |  |  |  | |  |
| Yes: | | | 45 (34) |  |  | 231 (94) |  |  |  | |  |
| Median time to start of oncological treatment^4^ (days and range): | | | 28 (8-144) | |  | 59 (20-187) | |  | <0.0001* | |  |
| ^1^Performance status, according to ECOG, Eastern Cooperative Oncology Group/WHO, ^2^Multilobular tumor, involving two or more cerebral lobes, ^3^Multifocal tumor, defined as at least two separate contrast-enhancing lesions, ^4^Waiting time from radiological diagnosis to start of first oncological treatment, *significant | | | | | | | | | |  |  |
