# Supplementary material for: Patterns of care and clinical outcome in assumed glioblastoma without tissue diagnosis: A population-based study of 131 consecutive patients
Source: PLoS One. 2020 Feb 13;15(2):e0228480. doi: 10.1371/journal.pone.0228480 (PMC7017992; doi:10.1371/journal.pone.0228480)
Supplement: S2 Table — (DOCX) [file pone.0228480.s002.docx]

**S2. De-identified minimal data set of the study population**

| **Pat number** | **Sex (1=M, 2=F)** | **Age in years** | **Age in days** | **Survival days** | **Survival months** | **Onc treatment (yes=1, no=0)** | **PS at diagnosis 0-4** |
| --- | --- | --- | --- | --- | --- | --- | --- |
| 1 | 1 | 85,6 | 31248 | 28 | 0,9 | 0 | 4 |
| 2 | 2 | 80,6 | 29445 | 151 | 5,0 | 1 | 0 |
| 3 | 2 | 84,8 | 30962 | 45 | 1,5 | 0 | 2 |
| 4 | 2 | 78,6 | 28706 | 68 | 2,3 | 0 | 4 |
| 5 | 2 | 76,3 | 27863 | 43 | 1,4 | 0 | 1 |
| 6 | 2 | 81,7 | 29836 | 104 | 3,5 | 0 | 2 |
| 7 | 1 | 64,1 | 23412 | 123 | 4,1 | 1 | 1 |
| 8 | 1 | 84,3 | 30777 | 629 | 21,0 | 0 | 3 |
| 9 | 1 | 82,1 | 29981 | 9 | 0,3 | 0 | 4 |
| 10 | 1 | 88,1 | 32194 | 172 | 5,7 | 0 | 1 |
| 11 | 1 | 78,7 | 28758 | 193 | 6,4 | 1 | 2 |
| 12 | 2 | 72,5 | 26489 | 385 | 12,8 | 1 | 1 |
| 13 | 1 | 68,5 | 25017 | 85 | 2,8 | 1 | 2 |
| 14 | 1 | 81,0 | 29593 | 15 | 0,5 | 0 | 4 |
| 15 | 2 | 90,5 | 33070 | 46 | 1,5 | 0 | 4 |
| 16 | 1 | 78,9 | 28821 | 280 | 9,3 | 1 | 1 |
| 17 | 2 | 72,5 | 26471 | 337 | 11,2 | 1 | 2 |
| 18 | 2 | 83,4 | 30468 | 340 | 11,3 | 1 | 2 |
| 19 | 1 | 67,1 | 24511 | 123 | 4,1 | 1 | 2 |
| 20 | 1 | 85,3 | 31168 | 52 | 1,7 | 0 | 3 |
| 21 | 1 | 68,0 | 24830 | 28 | 0,9 | 0 | 3 |
| 22 | 1 | 87,3 | 31887 | 76 | 2,5 | 0 | 2 |
| 23 | 1 | 85,5 | 31231 | 11 | 0,4 | 0 | 4 |
| 24 | 2 | 75,6 | 27619 | 192 | 6,4 | 1 | 1 |
| 25 | 1 | 73,4 | 26796 | 82 | 2,7 | 0 | 3 |
| 26 | 2 | 82,0 | 29957 | 25 | 0,8 | 0 | 3 |
| 27 | 1 | 52,8 | 19275 | 83 | 2,8 | 1 | 3 |
| 28 | 2 | 86,2 | 31488 | 129 | 4,3 | 0 | 2 |
| 29 | 1 | 81,3 | 29701 | 71 | 2,4 | 0 | 3 |
| 30 | 2 | 65,1 | 23767 | 150 | 5,0 | 1 | 2 |
| 31 | 2 | 79,7 | 29096 | 76 | 2,5 | 0 | 3 |
| 32 | 1 | 80,4 | 29383 | 249 | 8,3 | 0 | 2 |
| 33 | 2 | 81,9 | 29896 | 186 | 6,2 | 0 | 2 |
| 34 | 1 | 85,3 | 31160 | 406 | 13,5 | 1 | 0 |
| 35 | 1 | 85,8 | 31326 | 57 | 1,9 | 0 | 3 |
| 36 | 2 | 78,6 | 28723 | 55 | 1,8 | 0 | 4 |
| 37 | 2 | 78,3 | 28611 | 71 | 2,4 | 0 | 3 |
| 38 | 1 | 86,1 | 31430 | 86 | 2,9 | 0 | 3 |
| 39 | 2 | 79,6 | 29072 | 170 | 5,7 | 0 | 4 |
| 40 | 2 | 78,7 | 28742 | 492 | 16,4 | 1 | 1 |
| 41 | 1 | 76,8 | 28042 | 262 | 8,7 | 1 | 1 |
| 42 | 1 | 87,9 | 32108 | 85 | 2,8 | 0 | 3 |
| 43 | 1 | 58,4 | 21333 | 211 | 7,0 | 1 | 3 |
| 44 | 2 | 83,7 | 30587 | 201 | 6,7 | 0 | 2 |
| 45 | 2 | 75,8 | 27700 | 129 | 4,3 | 0 | 2 |
| 46 | 2 | 75,6 | 27619 | 192 | 6,4 | 1 | 1 |
| 47 | 2 | 78,7 | 28729 | 127 | 4,2 | 1 | 1 |
| 48 | 2 | 84,5 | 30859 | 68 | 2,3 | 0 | 3 |
| 49 | 1 | 83,3 | 30411 | 19 | 0,6 | 0 | 3 |
| 50 | 1 | 89,4 | 32668 | 92 | 3,1 | 0 | 2 |
| 51 | 1 | 83,1 | 30343 | 59 | 2,0 | 0 | 2 |
| 52 | 1 | 67,2 | 24555 | 267 | 8,9 | 1 | 0 |
| 53 | 2 | 84,7 | 30928 | 77 | 2,6 | 0 | 3 |
| 54 | 2 | 60,6 | 22133 | 41 | 1,4 | 0 | 2 |
| 55 | 1 | 75,6 | 27607 | 62 | 2,1 | 0 | 2 |
| 56 | 2 | 79,0 | 28839 | 235 | 7,8 | 1 | 1 |
| 57 | 1 | 81,3 | 29683 | 79 | 2,6 | 0 | 1 |
| 58 | 1 | 79,3 | 28976 | 135 | 4,5 | 0 | 2 |
| 59 | 1 | 72,3 | 26422 | 205 | 6,8 | 1 | 2 |
| 60 | 2 | 80,2 | 29280 | 182 | 6,1 | 0 | 4 |
| 61 | 1 | 79,8 | 29146 | 216 | 7,2 | 0 | 1 |
| 62 | 2 | 72,3 | 26396 | 219 | 7,3 | 1 | 2 |
| 63 | 2 | 81,2 | 29671 | 290 | 9,7 | 1 | 2 |
| 64 | 2 | 84,3 | 30777 | 102 | 3,4 | 0 | 1 |
| 65 | 1 | 58,8 | 21486 | 110 | 3,7 | 1 | 2 |
| 66 | 2 | 74,8 | 27326 | 177 | 5,9 | 1 | 0 |
| 67 | 1 | 81,2 | 29656 | 102 | 3,4 | 0 | 1 |
| 68 | 1 | 90,5 | 33046 | 12 | 0,4 | 0 | 2 |
| 69 | 1 | 71,5 | 26114 | 271 | 9,0 | 1 | 1 |
| 70 | 2 | 82,3 | 30064 | 112 | 3,7 | 0 | 2 |
| 71 | 2 | 71,7 | 26182 | 74 | 2,5 | 1 | 2 |
| 72 | 1 | 83,5 | 30490 | 64 | 2,1 | 0 | 1 |
| 73 | 1 | 67,5 | 24672 | 56 | 1,9 | 1 | 2 |
| 74 | 2 | 77,5 | 28294 | 238 | 7,9 | 1 | 1 |
| 75 | 1 | 78,0 | 28498 | 120 | 4,0 | 0 | 3 |
| 76 | 2 | 79,8 | 29133 | 208 | 6,9 | 0 | 2 |
| 77 | 1 | 74,9 | 27343 | 165 | 5,5 | 0 | 2 |
| 78 | 2 | 57,6 | 21046 | 134 | 4,5 | 1 | 1 |
| 79 | 2 | 73,6 | 26891 | 171 | 5,7 | 0 | 3 |
| 80 | 1 | 88,0 | 32142 | 213 | 7,1 | 0 | 2 |
| 81 | 2 | 75,7 | 27653 | 168 | 5,6 | 0 | 4 |
| 82 | 2 | 72,9 | 26616 | 154 | 5,1 | 1 | 1 |
| 83 | 1 | 76,7 | 28004 | 99 | 3,3 | 0 | 3 |
| 84 | 1 | 85,6 | 31281 | 90 | 3,0 | 0 | 3 |
| 85 | 2 | 87,1 | 31814 | 17 | 0,6 | 0 | 3 |
| 86 | 2 | 68,5 | 25023 | 229 | 7,6 | 1 | 0 |
| 87 | 1 | 73,5 | 26840 | 107 | 3,6 | 1 | 2 |
| 88 | 2 | 80,1 | 29239 | 135 | 4,5 | 0 | 1 |
| 89 | 2 | 75,2 | 27451 | 210 | 7,0 | 1 | 2 |
| 90 | 1 | 84,9 | 30997 | 49 | 1,6 | 0 | 3 |
| 91 | 1 | 75,4 | 27556 | 170 | 5,7 | 0 | 2 |
| 92 | 2 | 75,6 | 27608 | 100 | 3,3 | 0 | 3 |
| 93 | 1 | 83,1 | 30347 | 162 | 5,4 | 0 | 3 |
| 94 | 1 | 82,5 | 30128 | 36 | 1,2 | 0 | 3 |
| 95 | 1 | 80,0 | 29214 | 87 | 2,9 | 0 | 4 |
| 96 | 1 | 79,5 | 29038 | 10 | 0,3 | 0 | 4 |
| 97 | 2 | 85,9 | 31358 | 45 | 1,5 | 0 | 4 |
| 98 | 1 | 56,2 | 20528 | 351 | 11,7 | 1 | 1 |
| 99 | 1 | 63,9 | 23345 | 203 | 6,8 | 1 | 4 |
| 100 | 1 | 90,0 | 32856 | 194 | 6,5 | 0 | 1 |
| 101 | 2 | 81,7 | 29835 | 68 | 2,3 | 0 | 1 |
| 102 | 2 | 84,6 | 30886 | 7 | 0,2 | 0 | 3 |
| 103 | 1 | 88,5 | 32338 | 157 | 5,2 | 0 | 1 |
| 104 | 1 | 88,7 | 32384 | 25 | 0,8 | 0 | 2 |
| 105 | 1 | 89,9 | 32851 | 99 | 3,3 | 0 | 1 |
| 106 | 1 | 77,8 | 28418 | 65 | 2,2 | 1 | 2 |
| 107 | 2 | 65,2 | 23797 | 109 | 3,6 | 1 | 1 |
| 108 | 2 | 79,7 | 29122 | 91 | 3,0 | 0 | 4 |
| 109 | 1 | 83,0 | 30312 | 76 | 2,5 | 0 | 1 |
| 110 | 1 | 78,2 | 28567 | 50 | 1,7 | 0 | 2 |
| 111 | 1 | 86,3 | 31522 | 22 | 0,7 | 0 | 4 |
| 112 | 1 | 79,0 | 28838 | 132 | 4,4 | 1 | 2 |
| 113 | 2 | 82,1 | 29975 | 230 | 7,7 | 0 | 1 |
| 114 | 2 | 76,1 | 27811 | 113 | 3,8 | 0 | 2 |
| 115 | 1 | 78,0 | 28485 | 63 | 2,1 | 0 | 2 |
| 116 | 1 | 86,9 | 31751 | 117 | 3,9 | 0 | 1 |
| 117 | 1 | 73,2 | 26747 | 85 | 2,8 | 0 | 2 |
| 118 | 1 | 82,0 | 29954 | 274 | 9,1 | 1 | 0 |
| 119 | 2 | 72,8 | 26595 | 13 | 0,4 | 0 | 3 |
| 120 | 1 | 80,1 | 29242 | 87 | 2,9 | 0 | 3 |
| 121 | 2 | 73,8 | 26969 | 75 | 2,5 | 0 | 2 |
| 122 | 2 | 78,3 | 28605 | 419 | 14,0 | 1 | 2 |
| 123 | 1 | 76,3 | 27855 | 53 | 1,8 | 0 | 3 |
| 124 | 2 | 82,0 | 29935 | 126 | 4,2 | 0 | 4 |
| 125 | 1 | 78,5 | 28661 | 91 | 3,0 | 0 | 3 |
| 126 | 2 | 83,9 | 30642 | 100 | 3,3 | 0 | 3 |
| 127 | 2 | 76,9 | 28084 | 166 | 5,5 | 1 | 2 |
| 128 | 1 | 84,1 | 30706 | 567 | 18,9 | 1 | 0 |
| 129 | 1 | 82,9 | 30272 | 230 | 7,7 | 1 | 1 |
| 130 | 2 | 70,2 | 25638 | 84 | 2,8 | 1 | 2 |
| 131 | 1 | 84,4 | 30814 | 46 | 1,5 | 0 | 3 |
